# MiR-124 Regulates IQGAP1 and Participates in the Relationship Between Morphine Dependence Susceptibility and Cognition

**DOI:** 10.3389/fpsyt.2022.845357

**Published:** 2022-03-23

**Authors:** Jingjing Shi, Yong Chi, Xiaohong Wang, Yingjie Zhang, Lu Tian, Yao Chen, Chunwu Chen, Yong Dong, Hong Sang, Ming Chen, Lei Liu, Na Zhao, Chuanyi Kang, Xiaorui Hu, Xueying Wang, Qingxia Liu, Xuemin Li, Shuang Zhu, Mingxuan Nie, Honghui Wang, Liying Yang, Jiacheng Liu, Huaizhi Wang, Jia Lu, Jian Hu

**Affiliations:** ^1^Department of Psychiatry, The First Affiliated Hospital of Harbin Medical University, Harbin, China; ^2^The National Clinical Research Center for Mental Disorders, Capital Medical University & Beijing Key Laboratory of Mental Disorders, Beijing Anding Hospital, Capital Medical University, Beijing, China; ^3^Beijing Institute for Brain Disorders Center of Schizophrenia, Beijing Anding Hospital, Capital Medical University, Beijing, China; ^4^Shenyang Mental Health Center, Shenyang, China; ^5^Changchun Sixth Hospital, Changchun, China; ^6^Harbin University of Science and Technology, Harbin, China

**Keywords:** morphine dependence, mir-124, IQGAP1, addictive, cognition

## Abstract

**Background:**

Long-term excessive use of morphine leads to addictive diseases and affects cognitive function. Cognitive performance is associated with genetic characteristics.MiR-124 plays a critical regulatory role in neurogenesis, synaptic development, brain plasticity, and the use of addictive substances. As a scaffold protein, IQGAP1 affects learning and memory dose-dependent. However, the role of miR-124 and its target protein as potential addiction biomarkers and the impact on cognitive function have not been fully explored.

**Method:**

A total of 40 patients with morphine dependence and 40 cases of healthy people were recruited. We collected basic and clinical information about the two groups. The Generalized Anxiety Disorder Scale (GAD-7), Patient Health Questionnaire-9(PHQ-9), Montreal Cognition Assessment Scale (MoCA), Pittsburgh Sleep Quality Index (PSQI) were used to assess the severity of depression, anxiety, depressive symptoms, cognitive dysfunction, and sleep quality.

**Results:**

Compared to the control group, the morphine-dependent group had higher GAD-7, PHQ-9, PSQI scores, and more elevated miR-124 levels but lower MOCA scores and IQGAP1 levels. MiR-124, IQGAP1, the average intake last year were related to OASI scores.MiR-124, IQGAP1, PHQ-9 were associated with MOCA scores. In the multiple regression model, the levels of miR-124 and IQGAP1 were independent factors influencing the severity of morphine dependence. The level of miR-124 was an independent factor influencing the severity of cognitive impairment in patients with morphine dependence. In addition, the luciferase report confirmed that IQGAP1 mRNA is the direct target of miR-124.

**Conclusion:**

MiR-124 and its target protein IQGAP1 are involved in the regulation of addiction and cognitive function in patients with morphine dependence.

## Introduction

Over the past two decades, the abuse of opioids has led to a high mortality rate (1, 2). Among opioid drugs, morphine is considered one of the most effective analgesics for application in postoperative and cancer pain, the trouble about overuse and addiction it causes is rather intractable. Morphine and other opioid drugs can induce a broad spectrum of pharmacological activity. Occurring in the central nervous system, this generates a series of symptoms like disturbances in mood and promotes anxiety, depression, and cognitive impairments (3, 4). Frequent exposure to opioids also causes deficits in learning, memory, attention, reasoning, and impulse control (5). More explanations about how morphine and other opioid drugs affect cognitive function must be clarified.

MicroRNAs (miRNAs) are small endogenous non-coding RNAs that negatively regulate protein translation by binding to the 3′-untranslated regions (UTRs) of their target messenger RNAs (mRNAs). Many pieces of evidence show that miRNAs play a vital role in various physiological and pathological processes such as neurological diseases, mental diseases, addictive diseases, and cognitive impairment. MiR-124 is one of the most conserved and expressed neuron-specific miRNAs (6). It is abundantly expressed in the hippocampus, and has significant activity in neurons that differentiate and affect the generation, survival, and neuron generation, branching, excitation, and plasticity of cells (7). In terms of substance abuse, studies have found that long-term and overuse of cocaine and amphetamine can cause changes in miR-124 levels (8, 9). IQ motif containing guanosine triphosphatase activating protein 1 (IQGAP1), is a new protein that supports long-term memory. It is a 190 kDa scaffold protein that contains multiple domains and can interact with different targets (10). As an essential component of NMDAR multiprotein complexes, IQGAP1 is involved in the N-cadherin/cytoskeletal IQGAP1/Erk signaling pathway. It contributes to GluN1/GluN2A trafficking and facilitates IQGAP1-influenced memory formation. In animal research, IQGAP1 knockout mice exhibited impairments in fear conditioning, significantly lower surface NR2A, and impaired ERK activity compared to their wild-type littermates. They also performed marked long-term memory deficits accompanied by an impaired hippocampal long-term potentiation (LTP) (11). In addition, miR-124 and IQGAP1 are co-expressed in neuronal cells, suggesting that IQGAP1 may be a direct target of miR-124 in the brain (12).

Based on previous studies, we hypothesized that IQGAP1, as a direct target of miR-124, may be involved in regulating the addiction and cognitive function of patients with morphine dependence. Therefore, the first aim of this experiment was to explore whether miR-124 and IQGAP1 are susceptibility markers of morphine dependence. The second aim was to investigate whether miR-124 and IQGAP1 affect cognitive function. The final aim was to determine whether IQGAP1 was the target of miR-124.

## Methods

### Participants

From January 2021 to November 2021, 40 cases of morphine dependent patients (the morphine dependent group) were enrolled in Beijing Youan Hospital, Capital Medical University; 40 subjects of healthy people (the control group) were enrolled in Beijing Anding Hospital, Capital Medical University. Morphine dependent patients met the following criteria: (1) age of 18–45 years old; (2) diagnosis of Opioid Use Disorder Standard for DSM-5.The control group met the following criteria: (1) age of 18–45 years old; (2) there are no previous or current Axis I disorders, severe or unstable clinical diseases, neurological disorders, or any substance use 30 days before the study (self-report). Before all sample and data collection, participants were informed of the study's purpose and procedures, and signed an informed consent form. In addition, the Ethics Committee of Beijing Anding Hospital and Beijing Youan Hospital approved the trial.

### Clinical Assessment

Opioid Addiction Severity Inventory (OASI) was used to assess the severity of morphine addiction in patients. It consists of 4 subscales: physical dependence, psychological dependence, health harm, and social functioning harm. Questions were asked about past month heroin use. Each item was scored using a 4-point Likert scale, and the severity of opioid dependence was assessed by summarizing the item scores (13).

The Generalized Anxiety Disorder Scale (GAD-7) was used to assess the degree of anxiety in patients. GAD-7 asks how often people have suffered from the seven core symptoms of GAD in the past 2 weeks, with response options of “not at all,” “some days,” “more than half the days,” and “almost every day” (each option scored 0–3, total score 0–21). Researchers use GAD-7 as an indicator of treatment outcome in mixed anxiety and depression samples (14).

Patient Health Questionnaire-9(PHQ-9) was used to assess the severity of depressive symptoms. The PHQ-9 is a simple and validated self-rating scale for depressive disorders. It has good reliability and validity both as an aid in the diagnosis of depression and the assessment of symptom severity. The scale consists of 10 items, including nine symptom scales and one total functional rating. It is a 4-level scale, rated by the frequency of symptoms in the last 2 weeks. The total score range was 0–27, with higher scores being more severe for depressive symptoms.

Montreal Cognition Assessment Scale (MoCA) was used to screen for cognitive dysfunction. The MOCA has 11 examination items, including eight cognitive domains. The total score is 30, and a score of ≥26 is considered normal cognitive function, plus one if the number of years of education is ≤ 12, with higher scores indicating better cognitive function. The scale is highly sensitive, contains critical cognitive domains, has a short test time, is suitable for clinical application, and is more acute in screening for mild cognitive impairment.

Pittsburgh Sleep Quality Index (PSQI) was used to evaluate sleep quality. The PSQI was developed in 1989 by Dr. Buysse, a psychiatrist at the University of Pittsburgh, to assess sleep quality in the last month. The PSQI consists of 19 self-rated and five other-rated items. The 19th self-rated item and the 5th other-rated item are not involved in the scoring and consists of 7 components, each of which is scored of 0 to 3. The cumulative score for each component is the total PSQI score, which ranges from 0 to 2l. The higher the score, the worse the quality of sleep.

### Study Procedures

The subjects were evaluated for psychiatric diagnosis, drug use history, and clinical scales. They were recording the primary clinical information. At 6:00 am, the subjects were collected 5ML of peripheral venous blood on an empty stomach, and all blood samples were stored at a temperature of −20°C for subsequent testing.

#### Quantitative Measurement of miR-124, IQGAP1 MRNA

After extracting RNA from whole blood, use EntiLink™ 1st Strand cDNA Synthesis Kit (ELK Biotechnology, EQ003) to synthesize first-strand Cdna, and use EnTurbo™ SYBR Green PCR SuperMix Kit (ELK Biotechnology, EQ001) for synthesis Real-time fluorescent quantitative PCR detection. The mRNA primers were synthesized by Wuhan Jinkairui Biological Engineering Co., Ltd., using GAPDH as the internal control. Small nuclear RNA U6 snRNA was used as the internal control. Fold change analyses were performed following the 2^−Δ*ΔCt*^ method (6).

### Dual-Luciferase Reporter Assay

To verify whether miR-214 directly targets IQGAP1, firstly, constructing the pGL6-IQGAP1 vector. The IQGAP1 target gene was synthesized by ELK biotechnology company. The IQGAP1 was amplified by PCR and connected to the overlapping sequence of the vector, and then recombined with the digested vector. The relevant information is as follows:

IQGAP1 5′-3′ primer sequences:

IQGAP1 Forward: CCGTGTAAAGATCCGGTACCCAGAGAGACAATTCACTCCA.
IQGAP1 Reverse: CTCCTCGAGGATATCGGATCCAAAGTGTATGACTTTTTATC.
miRNA Sequence: CCGUAAGUGGCGCACGGAAU.

PCR was amplified by denaturation, annealing, and extension procedures. After 30 min of recombination reaction at 37°C, high-efficiency DH5a competent cells were used for transformation. After 1 day, single clones were picked for sequencing detection, and single colonies with correct sequencing were used for seed preservation and endotoxin-free plasmid extraction. Next, the cells are grouped and prepared, and the cells are grouped as follows: (a) NC; (b) pGL6-miR-IQGAP1- WT 3′UTR-+pRL-TK; (c) pGL6-miR-IQGAP1- WT 3′UTR+mimics-NC+ pRL-TK; (d) pGL6-miR-IQGAP1- WT 3′UTR+miR-124-3p+pRL-TK;(e) pGL6-miR-IQGAP1-mut-3′UTR + pRL-TK; (f) pGL6-miR-IQGAP1-mut-3′UTR+mimics-NC+ pRL-TK; (g) pGL6-miR-IQGAP1-mut-3′UTR+ miR-124-3p + pRL-TK. Subsequently, the preparation of the transfection complex was carried out, and the dual-luciferase reporter gene detection was carried out after transfection, in the case of using Renilla luciferase as the internal control, the RLU value of firefly luciferase assay was divided by the RLU value of Renilla luciferase assay.

### Statistical Analysis

Our study's statistical software package used for statistical calculations is the Statistical Program for Social Sciences (SPSS, version 22.0). We used the analysis of variance (ANOVA) and Chi-square test to compare the two groups' demographic data and clinical data. Spearman was used for correlation analysis, Bonferroni correction was used to adjust for multiple tests, and the Bonferroni-corrected indicators were included in the stepwise logistic regression, to determine the independent influencing factors that affect OASI score and MOCA score. Statistical significance was accepted when *P* < 0.05.

## Results

### Comparison of Demographic and Clinical Variables Between the Morphine Dependence Group and the Control Group

A total of 40 patients with morphine dependence were included. The average intake in the past year was 4,564 mg/week, and the median OASI score was 32 points. The demographic data and clinical variables of the two groups were compared, the results showed that compared with the control group, the GAD-7 score (*Z* = −6.499, *P* < 0.001), PHQ-9 score (*Z* = −7.621, *P* < 0.001), PSQI score was found to be increased in the morphine dependence group, the MOCA score (*Z* = −7.557, *P* < 0.001) was found to be decreased in the morphine dependence group; the level of miRNA-124 (*Z* = −3.017, *P* < 0.001) was higher than the control group, and the level of IQGAP1 (*Z* = −3.999, *P* < 0.001) was lower than the control group. The above indicators were corrected by Bonferroni (Bonferroni corrected *P* < 0.05/13 = 0.0038). There was no statistically significant difference among other indicators (Table 1).

**Table 1:** Comparison of demographic and clinical variables between morphine-dependent and control groups.

**Characteristics**	**Control group**	**Morphine dependent group**	***Z/X^**2**^***	***p***
Age (years)	41.5 (28.00, 51.25)	36.00 (29.00, 47.00)	−0.539	0.590
BMI	21.5 (20.15, 23.18)	22.25 (20.49, 26.73)	−1.830	0.067
GAD-7 score	0.00 (0.00, 0.75)	9.00 (3.75, 14.75)	−6.499	<0.001
PHQ-9 score	0.00 (0.00, 0.75)	13.50 (7.25, 18.75)	−7.621	<0.001
MOCA score	30.00 (30.00,30.00)	26.00 (24.00, 29.00)	−7.557	<0.001
PSQI score	0.00 (0.00, 0.00)	16.50 (13.00, 18.00)	−7.932	<0.001
miRNA-124	0.40 (0.18, 0.69)	0.99 (0.45, 1.37)	−3.017	0.003
IQGAP1	1.68 (1.03, 2.28)	0.97 (0.60, 1.34)	−3.999	<0.001
OASI score	-	32.00 (25.75, 37.00)		
Average intake in the past year	-	4564.00 (2650.00, 7000.00)		
Male, *N* (%)	35 (87.5%)	35 (87.5%)	0.000	1.000
Education, *N* (%)			2.656	0.448
Junior high school	10 (25.0%)	11 (27.5%)		
Senior high school	14 (35.0%)	17 (42.5%)		
College	16 (40.0%)	11 (27.5%)		
Postgraduate	0 (0.0%)	1 (2.5%)		
Smoking	33 (82.5%)	35 (87.5%)	0.392	0.531
With physical disease	19 (47.5%)	14 (35.0%)	1.289	0.256

*GAD-7 Score, The Generalized Anxiety Disorder Scale Score; PHQ-9 Score, Patient Health Questionnaire-9 Score; MOCA Score, Montreal Cognition Assessment Scale Score; PSQI Score, Pittsburgh Sleep Quality Index Score; OASI Score, Opioid Addiction Severity Inventory Score*.

### Correlation Analysis of OASI and MOCA Total Scores in Patients With Morphine Dependence

The results of correlation analysis between OASI scores and MOCA scores in patients with morphine dependence found that the miR-124 levels (*r* = −0.384, *P* < 0.001), the IQGAP1 levels (*r* = −0.597, *P* < 0.001), GAD-7 scores (*r* = 0.377, *P* = 0.016), the average intake in the past year (*r* = 0.508, *p* = 0.001) were related to OASI scores, but GAD-7 scores were not corrected by Bonferroni (Bonferroni corrected *P* < 0.05/11 = 0.0045). The miR-124 levels(*r* = −0.578, *P* < 0.001), the IQGAP1 levels (*r* = 0.486, *P* = 0.001), GAD-7 scores (*r* = −0.361, *P* = 0.022), PHQ-9 scores (*r* = −0.499, *P* = 0.001), the average intake in the past year (*r* = −0.382, *P* = 0.015) were related to the MOCA scores, but GAD-7 scores and the average intake in the past year did not pass Bonferroni correction (Bonferroni corrected *P* < 0.05/11 = 0.0045) (Table 2).

**Table 2:** Correlation analysis of OASI and MOCA total score in morphine-dependent patients^a^.

	**1**	**2**	**3**	**4**	**5**	**6**	**7**	**8**	**9**	**10**	**11**	**12**
1. OASI	1	−0.384	0.605	−0.597	0.291	0.377	0.228	−0.077	0.223	0.153	0.013	0.508
2. MOCA	0.014	1	−0.578	0.486	−0.092	−0.361	−0.499	0.065	−0.026	0.167	0.003	−0.382
3. miRNA-124	<0.001	<0.001	1	−0.530	0.090	0.337	0.446	−0.315	−0.036	−0.241	−0.010	0.401
4. IQGAP1	<0.001	0.001	0.000	1	0.082	−0.288	−0.340	0.039	−0.174	0.097	−0.239	−0.416
5. BMI	0.077	0.581	0.589	0.625	1	−0.217	0.050	−0.108	−0.082	0.114	−0.021	0.228
6.GAD-7	0.016	0.022	0.033	0.072	0.191	1	0.278	0.150	0.217	0.189	−0.062	0.374
7.PHQ-9	0.158	0.001	0.004	0.032	0.765	0.082	1	−0.106	−0.013	−0.247	0.236	0.365
8.PSQI	0.636	0.692	0.048	0.809	0.520	0.356	0.513	1	0.231	0.178	−0.036	−0.094
9.Gender	0.166	0.871	0.825	0.284	0.626	0.179	0.936	0.152	1	0.095	−0.086	−0.062
10. Age	0.347	0.304	0.134	0.550	0.497	0.243	0.124	0.272	0.560	1	−0.043	0.049
11. Smoking	0.936	0.984	0.952	0.137	0.899	0.702	0.142	0.824	0.599	0.794	1	0.194
12. Average intake in the past year	0.001	0.015	0.010	0.008	0.168	0.017	0.021	0.564	0.702	0.765	0.230	1

*GAD-7 Score, The Generalized Anxiety Disorder Scale Score; PHQ-9 Score, Patient Health Questionnaire-9 Score; MOCA Score, Montreal Cognition Assessment Scale Score; PSQI Score, Pittsburgh Sleep Quality Index Score; OASI Score, Opioid Addiction Severity Inventory Score*.

*^a^Correlation matrix: each cell in the table shows the correlation between two variables. The line of 1 s going from the top left to the bottom right is the main diagonal. The number in each cell on the upper right part of the diagonal represents the correlation coefficient (r), while and the number in each cell on the lower left part is the corresponding p value, which is a mirror image of those above the diagonal*.

### Independent Influencing Factors of OASI Score in Patients With Morphine Dependence

Including meaningful indicators in the correlation analysis with the OASI total score in the multiple linear regression model, the results showed that the miR-124 level (*B* = 3.880, *P* = 0.012) and the IQGAP1 level (*B* = −5.883, *P* = 0.026) were independent factors influencing the severity of morphine dependence in patients with morphine dependence (Table 3).

**Table 3:** Linear regression analysis: analysis of independent influencing factors of OASI score.

**Variables**	**B**	**Std.error**	**β**	***p***	**95% CI**
miRNA-124	3.880	1.462	0.385	0.012	0.915 to 6.844
IQGAP1	−5.883	2.534	−0.346	0.026	−11.022 to 0.743
Average intake in the past year	3.440 × 10^5^	0.000	0.078	0.558	0.000 to 0.000152

*OASI Score, Opioid Addiction Severity Inventory Score*.

### Independent Influencing Factors of MOCA Score in Patients With Morphine Dependence

Including meaningful indicators in the correlation analysis with the MOCA score into the multiple linear regression model, the results showed that the level of miR-124 (*B* = −1.618, *P* = 0.007) was an independent factor influencing the severity of cognitive impairment in patients with morphine dependence (Table 4).

**Table 4:** Linear regression analysis: analysis of independent influencing factors of MOCA score.

**Variables**	**B**	**Std.error**	**β**	***p***	**95% CI**
miRNA-124	−1.618	0.565	−0.423	0.007	−2.764 to 0.471
IQGAP1	1.420	0.972	0.220	0.153	−0.552 to 3.392
Average intake in the past year	−0.081	0.062	−0.180	0.199	−0.205 to 0.044

*MOCA Score, Montreal Cognition Assessment Scale Score*.

### Bioinformatics Software Predicts That IQGAP1 Is a Potential Target Gene of miR-124

**Figure 1:**
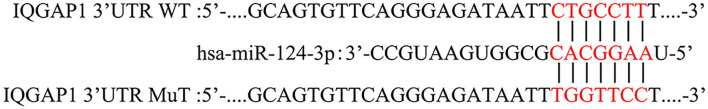
Bioinformatics software predicts that IQGAP1 is a potential target gene of miR-124. The binding sites of Mir-124 to IQGAP1.

The binding of miR-124 and IQGAP1 was analyzed by bioinformatics, and the results are shown in Figure 1. The results showed that miR-124 and IQGAP1 have complementary sequences that directly bind (Figure 1).

### Dual-Luciferase Reporter Gene to Detect the Binding of miR-124 and IQGAP1

The dual-luciferase reporter gene detects the binding of miR-124 and IQGAP1. First, in the NC group and Mimics-NC group, there was no statistically significant difference in luciferase activity between IQGAP1WT and IQGAP1Mut (*P* > 0.05). In addition, in the miR-124-3p mimic group, the luciferase activity of IQGAP1WT was significantly lower than that of IQGAP1Mut, and the difference was statistically significant (*P* < 0.05). The above result indicates that miR-124-3p directly binds with IQGAP1 (Table 5).

**Table 5:** The dual-luciferase reporter gene detects the binding of miR-124 and IQGAP1.

**Group**	**IQGAP1WT(***x*±*s***)**	**IQGAP1Mut(**x±*s***)**
NC	1.15 ± 0.1	1.15 ± 0.15
Mimics-NC	1.05 ± 0.11	1.1 ± 0.04
miR-124-3p mimics	0.46 ± 0.03	1.1 ± 0.16

*In the miR-124-3p mimic group, the luciferase activity of IQGAP1WT was significantly lower than that of IQGAP1Mut, wt, wild type; mut, mutant*.

## Discussion

This is the first time the mood, addiction, and cognitive function of miR-124 and its target protein in morphine-dependent patients has been studied. Our results show that morphine-dependent patients had higher GAD-7 scores, PHQ-9 scores, PSQI scores, and lower MOCA scores. They also had higher miR-124 levels and lower IQGAP1 levels. MiR-124 and IQGAP levels may predict the severity of dependence in morphine-dependent patients. MiRNA-124 levels may affect the degree of cognitive impairment in morphine-dependent patients.

The mechanisms related to the susceptibility of morphine-dependent patients and the emotional and cognitive impairment caused by morphine have not been fully revealed. MiRNAs and their target proteins may play an important role; miR-133, miR-146a, and miR-101 have been studied in the past. MiR-124 was found to be elevated in morphine-dependent patients in our study. This may be due to the activation of the NF-κB pathway by morphine, resulting in P65 binding to the promoter of miR-124 and promoting the transcription of miR-124. Qiu et al. found that acute morphine treatment could temporarily up-regulate the expression of P65 cells and then initiate the expression of miR-124 (15). The level of IQGAP1 decreased may be related to the conserved binding site of miR-124 in the 3′untranslated region (UTR) of IQGAP1. Our research and Fan et al. showed that induced mutations in the 3′untranslated region of IQGAP1 led to miR-124-mediated luciferase activation (16). This suggests that the miR-124 binding site in IQGAP1 is related to the down-regulation of IQGAP1. The increase of GAD-7 score, PHQ-9 score, and PISQ score in morphine-dependent patients may be related to morphine-induced anxiety and depression. The altered expression of dopamine receptors in the reward system may be related to morphine-induced anxiety. Vousooghi et al. showed that morphine-treated male offspring exhibited more anxiety-like behaviors and significantly increased D1 and D5 dopamine receptors in the prefrontal cortex and nucleus accumbens. Hippocampal D5 and D2 receptors decreased. The expression of the D4 dopamine receptor was raised in the striatum and hippocampus and decreased in the prefrontal cortex (17). In addition, some functional studies have also explored the causal relationship between miRNA expression and anxiety and depression-like behaviors. In animal models, the observed up-regulation or down-regulation of candidate miRNAs at important nodes of anxiety neural circuits can regulate anxiety-related behaviors. These identified miRNAs are related to specific neurotransmitter/neuromodulating signals, neurotrophic factor expression, synaptic plasticity, and stress regulation/hypothalamic-pituitary-axis function (18). Yang et al. found that knocking down miR-124 can improve the depression-like behavior of rats, which may be related at least in part to the up-regulation of CREB1 and BDNF expression in the hippocampus (19), under the influence of anxiety and depression symptoms, sleep quality declines. All in all, these data showed that miR-124 plays a vital role in genetic markers and symptoms of anxiety and depression in morphine dependence.

In this study, the OASI score is correlated with miR-124, IQGAP1, and morphine intake in the past year, and the levels of miR-124 and IQGAP1 are independent factors influencing the severity of morphine dependence in morphine-dependent patients. The basic mechanisms of opioid dependence and tolerance are complex changes in the levels of cells, synapses, and circuits in the central nervous system, as well as receptor phosphorylation, signal transduction, multimerization, etc. The enhancement of tolerance in the body also promotes the use of drugs, thereby increasing the formation of addictive behaviors. The administration of morphine can cause changes in the expression levels of multiple miRNAs in nerve tissues or cells. The miRNAs regulation model is transcription degradation or translation inhibition; its changes will affect the constitutive suppression of genes, which is essential for maintaining addictive behaviors (1). Studies have pointed out that after exogenous miR-124 supplementation *in vitro* (20), the neuronal differentiation level and glutamate transporter expression of human neural progenitor cells increase. Neuropathic pain and bone cancer pain-induced reduction of miR-124 in the brain and spinal cord of rats triggers microglial activation, leading to persistent hyperalgesia, which can be prevented by intrathecal administration of miR-124, so we speculate that miR-124 may be involved in the formation of morphine tolerance (21, 22). In other studies on the use of addictive substances, it has been found that the increase in peripheral blood miR-124 in cocaine-addicted women may be related to metabolism. In the research of alcohol use disorder, the rise of miR-124 can affect the alcohol intake behavior of mice by adjusting the HPA axis (23). Neurogenic differentiation (NeuroD) is critical for the development of both the central nervous system and the endocrine system. NeuroD is an important transcription factor during neurogenesis in the subgranular region of the adult hippocampus, regulating neural stem cell differentiation and migration. NeuroD also helps stabilize existing brain circuits and supports the formation of new circuits. Studies have found that miR-124 may regulate opioid addiction by affecting NeuroD-related pathways (24), and miR-124 interacts with the binding site of NeuroD1, which negatively regulates the expression of the preneural marker NeuroD1 (25). Morphine tolerance is an adaptive process thought to result from complex alterations in μ-opioid receptors (MORs) at the molecular level as well as at the synaptic, cellular and circuit levels, both in the peripheral and central nervous systems, where MORs are downregulated and neural adaptation may be the main mechanism of morphine tolerance (26). However, there are few independent studies on the function of miR-124 on MORs. Previous studies on the relationship between MORs and miRNAs found that miR-23b can complementarily bind to the 3′-UTR of MOR mRNA and reduce the expression of MOR at the post-transcriptional level. *In vitro*, chronic morphine exposure increased the expression of miR-339-3p in mouse hippocampal neurons, by binding to the 3′-UTR-specific sequence partially reversed by the miR-339-3p inhibitor, leading to the destabilization and degradation of MOR mRNA. However, it has also been reported that miR-16 can attenuate the translation of MOR mRNA, and morphine can upregulate MOR levels by inhibiting the expression of miR-16, but this finding originates from a study of CEM 174 cells (a lymphocyte lineages), but not on the nervous system (27). Our study found that patients' morphine addiction severity scores were positively correlated with miR-124, which led to speculation that long-term chronic morphine intake up-regulated the expression of miR-124 and that miR-124 was partially complementary to and bound to the 3′UTR of MORs mRNA. Thus, the translation of MORs was stopped, resulting in a decrease in MOR biosynthesis, aggravation of morphine tolerance, and an increase in morphine intake and dependence. As a direct target and influencing factor of miR-124, IQGAP1 also has a certain relationship with addiction and dependence. Sun et al. found that in primary rat and human cardiomyocytes, Methamphetamine (METH) exposure decreased the expression of primary rat cardiomyocytes and the downstream protein IQGAP1 *in vivo* (9). Studies have found that local translational control in the spine is a powerful mechanism for regulating morphological and functional plasticity; miRNAs are involved in dendritic spine morphogenesis and development and addiction (28). Chronic morphine treatment causes the dendritic spines of the hippocampal neuron culture to collapse. Because overexpression of Rac1 can induce the formation of dendritic spines, IQGAP1 can bind to Rac1 as a junction (integrating receptor signals) and a node (diversifying signals to multiple out-puts), improve dendritic spine collapse, adjust dendritic morphology, reshape actin cytoskeleton, affect synapse formation, adjust the sensitivity of reward pathways, change neuronal plasticity, and then affect the formation of morphine dependence (29). However, due to limited research on substance-dependent genes and their target proteins, more mechanisms remain to be discovered. In general, combined with our findings that miR-124 and IQGAP1 are involved in regulating the addiction and tolerance of morphine-dependent patients, the meaningful indicators in the OASI correlation analysis were incorporated into the multiple linear regression model, and it was found that miR- 124 and IQGAP1 are not only related to the severity of morphine dependence, but also can be used as independent influencing factors to affect the severity of morphine dependence in patients with morphine dependence, and are not affected by other related factors, both can be used as markers of the nervous system and are involved in the formation of morphine addiction, tolerance and dependence.

In recent years, the epigenetic mechanism of learning and memory has been a hot spot in cognition-related research. Previous studies have shown that both IQGAP1 and miRNAs expressed in the brain are involved in human learning and memory. The disorder of miRNAs function may be related to the occurrence or progression of neurodegenerative diseases. Our study found that miR-124 and IQGAP1 are associated with the total score of MOCA. The level of miR-124 is an independent factor influencing the severity of cognitive impairment in patients with morphine dependence. This indicates that IQGAP1, as one of the target proteins of miR-124, can regulate and participate in cognitive function through the change of miR-124 level and other related mechanisms. MiR-124 can not only affect the cognitive level of patients together with its target protein, but also act as an independent factor affecting the patient's cognition. Studies found that miR-124 was predicted to control important target genes involved in neuronal apoptosis and neuronal stress-induced adaptation. The decline of cognitive function is also considered to be related to cell dysfunction and the increase of apoptotic factors. The overexpression of some miRNAs (such as miR-34, etc.) is involved in reducing the apoptosis rate of neurons, reducing cell dysfunction, and playing a neuroprotective role (30). Zhao et al. demonstrated that miR-124 exerts its neuroprotective effect on sevoflurane by targeting Capn4 and NF-κB signaling pathways, reducing hippocampal neuronal apoptosis (31). Hassouna et al. suggest that recombinant human erythropoietin (EPO) improves cognitive ability in neuropsychiatric disorders, which is associated with miR-124. In cultured nerve spheres, they found that EPO stimulates miR-124, related to late neuronal differentiation (32). MiR-124 may play a vital role in the normal prefrontal cortex. Kozuka et al. found that miR-124 dosing regulates prefrontal cortex function by inhibiting the Drd2 pathway (33). Neural function in the central nervous system is closely related to signal transduction; the specific cellular functions of signal transduction pathways depend to a large extent on the regional regulation of scaffold proteins. The N-cadherin/IQGAP1/Erk-2 signaling pathway affects cognition, emotion, and motivational behavior. Mice lacking the IQGAP1 gene have significantly reduced NR2A levels on the surface and impaired ERK activity, and the reduction in the number of the spine in IQGAP1 knockout mice is region-specific. The hippocampus and lateral amygdale that affect memory and emotion are the most affected, showing long-term potentiation (LTP) damage (11). Yang et al. found that IQGAP1 binding site polymorphism with miR-124 can influence human cognitive performance. They concluded that individuals carrying the derived T-allele had higher IQGAP1 expression in the brain than their ancestral A-allele carriers (6). Overall, these results demonstrated that miR-124 and its target protein IQGAP1 are involved in regulating cognitive function in patients with morphine dependence.

The current research is helpful to understand the pathogenesis of morphine dependence based on genetics, and the level changes of miRNAs and their target protein can also be used as targets for the diagnosis and treatment of morphine dependence in the future. But our research also has limitations. First, the number of enrolled cases is small, and the result does not rule out the possibility of false positives. The number of cases needs to be increased. Secondly, we only conducted a cross-sectional study, and it is not clear whether there are any changes in blood indicators in patients with morphine dependence. Moreover, when we collect patients, we focus on the abuse of morphine, but people who use addictive substances are often prone to smoking, drinking, and other problems. Whether these factors impact the results is also an issue that needs attention. In conclusion, our research found that compared with the control group, the expression of miR-124 and IQGAP1 in morphine-dependent patients is significantly different. The levels of miR-124 and IQGAP1 are correlated with anxiety and depression symptoms, miR-124 and its target protein IQGAP1 are involved in regulating addiction and cognitive function in patients with morphine dependence.

## Data Availability Statement

The original contributions presented in the study are included in the article/supplementary material, further inquiries can be directed to the corresponding authors.

## Ethics Statement

The studies involving human participants were reviewed and approved by Ethics Committee of the First Affiliated Hospital of Harbin Medical University. The patients/participants provided their written informed consent to participate in this study. Written informed consent was obtained from the individual(s) for the publication of any potentially identifiable images or data included in this article.

## Author Contributions

JH and XiW: designed this research and revised the manuscript. JS: designed this research, performed the statistical analysis, and wrote the manuscript. YChi, YZ, and LT: collected data. YChe, CC, YD, HS, MC, LL, NZ, CK, XH, QL, XL, SZ, MN, HoW, LY, JLi, HuW, and JLu: organized the data. XuW: performed the statistical analysis. All authors contributed to the article and approved the submitted version.

## Funding

This study was supported by the National Key Research and Development Program of China (No. 2018YFC1314400, 2018YFC1314402).

## Conflict of Interest

The authors declare that the research was conducted in the absence of any commercial or financial relationships that could be construed as a potential conflict of interest.

## Publisher's Note

All claims expressed in this article are solely those of the authors and do not necessarily represent those of their affiliated organizations, or those of the publisher, the editors and the reviewers. Any product that may be evaluated in this article, or claim that may be made by its manufacturer, is not guaranteed or endorsed by the publisher.
